# Function of Tetra (4-Aminophenyl) Porphyrin in Altering the Electronic Performances of Reduced Graphene Oxide-Based Field Effect Transistor

**DOI:** 10.3390/molecules24213960

**Published:** 2019-10-31

**Authors:** Shihui Hu, Yunfang Jia

**Affiliations:** College of Electronic Information and Optical Engineering, Nankai University, Tianjin,300350, China; 1120170101@mail.nankai.edu.cn

**Keywords:** reduced graphene oxide, porphyrin, field effect transistor, tetra (4-aminophenyl) porphyrin

## Abstract

Porphyrin functionalized reduced graphene oxide (rGO) is attractive for multi-disciplinary research studies, and its improvements for an rGO-based field effect transistor (rGO-FET) were exploited to realize ultrasensitive biochemical and clinical assay. Although it was believed that the hybrids of porphyrin and rGO can make positive impacts on the rGO-FET’s electronic performances, the understandings of its functions are still piecemeal. Herein, the reduced mixtures of tetra (4-aminophenyl) porphyrin (TAP), GO (TAP-rGO), and the FET channeled by them are examined to throw a light on the possible approaches through which TAP affects rGO’s quality and its carrier mobilities. A TAP-caused game relationship is established by deliberating about the results of the intentionally altered experimental conditions, including TAP contents and the overmixing pretreatment. The p-type doping deduction for the right-shifted ambipolar transfer characteristic curves is evidenced by X-ray photoelectron spectroscopy (XPS). The problems posed by the TAP-induced FET features’ improvement, regression, and deterioration are clarified by the integrated proofs from Raman fingerprints, the amide and carboxyl groups’ changing trajectory found by C1s XPS core spectra, and the enlarged few-layer graphene morphology from atomic force microscope and transmission electron microscope. We hope that this effort will provide some constructive recommendations for producing low-cost graphene derivatives and promoting their applications in FET-like electronic components.

## 1. Introduction

Reduced graphene oxide (rGO) is one of the welcomed two-dimensional and biofunctional materials for field effect transistor (FET)-type biosensors to fulfill the clinical detections [1,2], due to its balancing talent to recover the destroyed graphene basal plane in graphene oxide (GO) and preserve part of the oxygen-containing groups on it [3]. As a conductive channel material, research on the contact resistance between graphene and metal to improve the performance of the field effect transistor has achieved corresponding results [4,5]. In addition, the demands for high carrier mobility and luxuriant functional groups stimulated lots of efforts, such as the heating-assisted spray method to produce large-scale rGO film [6], the interlayered quantum dots to elevate carriers mobilities [7], and so on. Comparatively speaking, porphyrin-functionalized chemically rGO may provide an inspiring solution for fabricating low-cost and biocompatible FET-type sensors, not only because of the economical merit inherent in its total liquid process, but also the promising biocompatibility [8] and biocatalysis in the oxygen reduction reaction (ORR) [9].

The strong interactive activities between porphyrin and rGO were found nearly a decade ago, in both their liquid and film states, including not only the ground-state and excited-stated interactions, but also the charge transfer from porphyrin to rGO [10]. At the same time, the increased conductivity of rGO was achieved by the porphyrin-stabilized reduction process [11]. Later, in the nonlinear optical study of a GO and tetra (4-aminophenyl) porphyrin (TAP) hybrid, an electron and/or energy transfer from photoexcited TAP to its covalently linked GO moiety was also manifested [12]. Motivated by those fundamental studies, more interesting studies about the physicochemical mechanism between porphyrin and graphene [13], as well as the applications of their hybrids, have been reported. For instance, the decorated rGO-based field effect transistor (rGO-FET) by the composite of Fe(III) meso-tetra (4-carboxyphenyl) porphyrin (FeTCP) and rGO (FGPCs) was reported to realize the ultrasensitive NO detection under a lower driving voltage (V_DS_ = 0.1 V) [14], in which the mechanism for the increased sensitivity was ascribed to the charge transfer between porphyrin and rGO, just in accordance with the recent publication about porphyrin-facilitated hydrogen evolution reaction (HER) at the solution–graphene interface [13]. Moreover, based on the similar HER principle, the hybrid of TAP and rGO (TAP-rGO) was also exploited as the channel material in bio-FET and applied for the detection of circulating tumor cells [2].

According to the summary of those works, it could been perceived that porphyrin can provide modulations in the process of rGO preparation and/or carriers’ transfer in FET: in the raw material preparation stage, it can stabilize GO dispersion [11], or form amide linkages with GO fragments [12]; in the chemical reduction stage, the porphyrin-stabilized GO dispersion may be reduced more deeply [11], and its catalysis in ORR is also helpful for the deoxygenation process [9]; in the working stage of FET, surface-modified porphyrin can facilitate charge transfer at the solid–liquid interface [13], and the similar HER effect is also found in porphyrin–rGO hybrid decorated [14] and channeled [2] FETs. Although it was believed that the hybrid of TAP and rGO can have a positive influence on FET’s electronic performances, the study of TAP’s function from the point of view of FET has not been found. 

**Figure 1:**
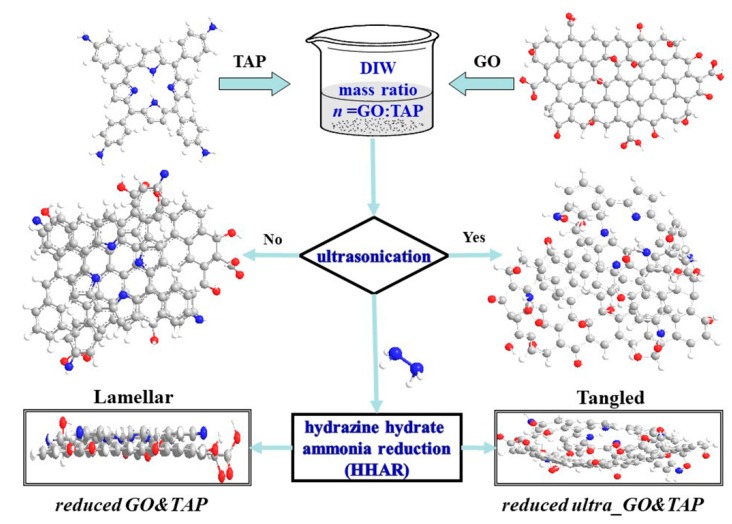
The experimental protocol for preparing tetra (4-aminophenyl) porphyrin (TAP) and reduced graphene oxide (rGO) hybrid for the channel film of the rGO-based field effect transistors (rGO-FETs). The precursors are the mixture of GO and TAP (GO&TAP) in deionized water with mass ration (*n* = GO:TAP) and the ultrasonically treated one (named as ultra_GO&TAP), respectively. After being reduced by the similar hydrazine hydrate and ammonia reduction (HHAR), their products are named as reduced GO&TAP, reduced ultra_GO&TAP, respectively.

Herein, to have a knowledge of TAP’s role in TAP-rGO channeled FETs, the mixing degree of TAP in its hybrid with rGO is altered by changing the mass ratios of GO to TAP (*n*) and applying the pretreatment of water-bath ultrasonication to its reducing precursor (GO&TAP), as depicted in Figure 1. In this figure, GO is prepared by the modified Hummers method, while the conventional hydrazine hydrate and ammonia reduction (HHAR) process is utilized to reduce GO and its mixtures with TAP. By this protocol, three kinds of the reduced products are synthesized, which are the reduced GO and TAP mixture (reduced GO&TAP), and the reduced ultrasonically treated GO&TAP (reduced ultra_GO&TAP), as well as pure rGO. The comparison and integrated analyses of the material’s characterizations and FETs’ electronic performances are conducted to throw light on the possible approaches through which TAP impacts the process of reducing GO, restoring the distorted GO lattice, and then ultimately changing the electronic performances of TAP-rGO-based FETs.

## 2. Results

### 2.1. Electronic Performance of FETs

**Figure 2:**
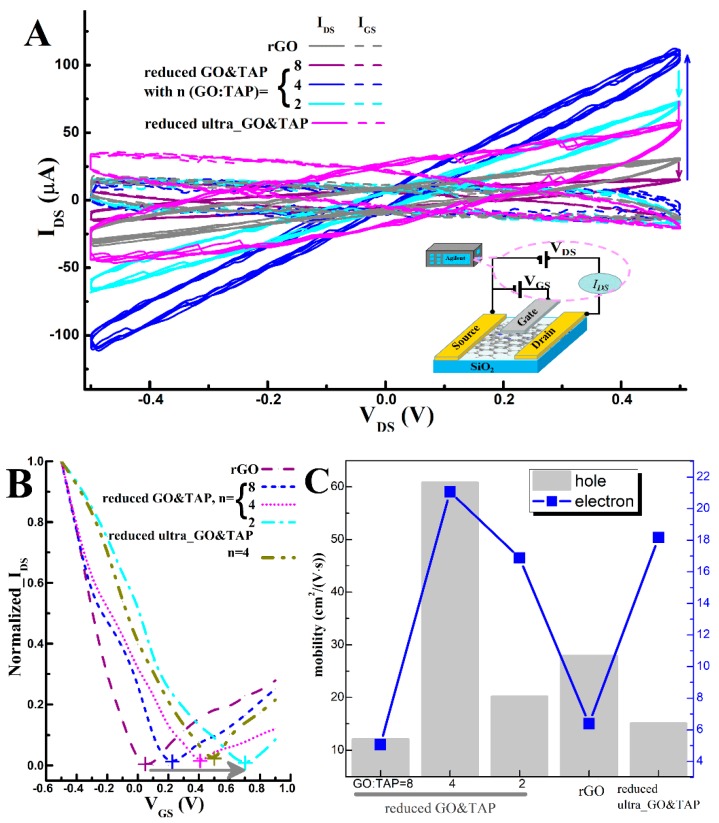
The electronic features of FETs channeled by different reduced products, which are reduced GO&TAP with a mass ratio of GO:TAP (*n*) = 8, 4, 2, reduced ultrasonic pretreated GO&TAP (ultra_GO&TAP, *n* = 4) and pure rGO. (**A**) Output features. FET’s structure and detection system are depicted in the inset. I_DS_ is the current between the drain and source, V_GS_ and V_DS_ are the voltages of the gate and drain, in reference to the source. V_DS_ is swept from −0.5 V to + 0.5 V for 10 rounds, while V_GS_ is controlled at 0.2 V. (**B**) The normalized transfer characteristic curves (TCCs), the measured data are presented in Figure S2 (V_DS_ = 0.5 V). (**C**) The calculated carriers’ mobilities for the samples.

The electronic features of the as-prepared TAP-rGO-based FETs are examined and presented in Figure 2. Firstly, it is found the output currents (I_DS_) are altered with the addition of TAP, but the changing of I_DS_ is irregular, as shown by the arrows in Figure 2A. At the same bias voltages (V_GS_), the highest output currents I_DS_ and the lowest leaking current from Gate (I_GS_) are observed by the reduced GO&TAP with *n* = 4 (blue curves); however, for its ultrasonically treated counterpart (the magenta curves), I_DS_ curves are lowered, and I_GS_ curves are raised. For most of the TAP-rGO-based FETs, higher I_DS_ values could be measured than rGO ones; however, when TAP contents are increased from zero (rGO, dark yellow curves) to 1/8 of GO (*n* = 8, purple curves), decreased I_DS_ is found. In addition, in Figure 2A, the almost zero leaking currents from the gate (I_GS_, dash curves) are measured by most of the devices, except for the reduced ultra_GO&TAP. The increased I_GS_ indicates the carriers that should be confined in the transversal channel region, fled away in the vertical direction, and driven by V_GS_. The definition for directions is provided in Figure S1. We would like to ascribe it to the tangled rGO and TAP structure, as depicted in Figure 1. More discussions will be presented in the following section, aided by Raman and X-ray photoelectron spectroscopy (XPS) results.

Meanwhile, the transfer characteristic curves (TCCs) (Figure 2B) indicate that all of the samples have the typical ambipolar characteristic. The close to zero charge neutral point (CNP, the crosses “+” in Figure 2B) in TCC of pure rGO (purple) is similar to the reported results in the literature [1], but is different from [14], which means that there is less oxygen residue. In terms of the curves’ contour, the scattering cross-sections of charged impurities for electrons and holes [15] and the contact of graphene with electrodes [16] are both likely to cause their asymmetry. At the interface of the metal Cu and graphene, electrons move from the low work function side (Cu, about 4.65 eV) to the graphene channel (about 4.8 eV), while the holes move in reverse. For the h-branch, there are more holes in the graphene channel; it is helpful to enhance the carriers’ transport at the Cu–graphene interface. On the contrary, for the e-branch, there are more electrons than holes, which is adverse to the interface carriers’ movement. Thereby, the currents (I_DS_) changing tendencies in the h-branch and e-branch are asymmetric. It is also found that the locations of the curves are right-shifted from rGO (gray curves) to reduced GO&TAP (blue, magenta, and cyan curves are corresponding to *n* = 8, 4, 2, respectively) and comply with the p-type doping effect [1], which is deduced to be caused by the N elements in TAP. Meanwhile, the differences of the TCC’s moving direction in comparison with FGPC-decorated rGO-FET (which was left-shifted) could be ascribed to the opposite charges in FeTCP (negative) and TAP (positive) [14].

For the same TAP content (*n* = 4), the right-side movement of the dark-yellow TCC (reduced ultra_GO&TAP, *n* = 4) overpassed its counterpart (magenta, reduced GO&TAP, *n* = 4); the ultrasonication increasing the TAP mixing degree may contribute to this movement. This deduction will be illustrated according to the XPS results in the next section.

A similar investigation for the relation between I_GS_ and V_GS_ is also executed at the controlled V_DS_ (0.5 V) and presented in Figure S3, in order to have more understanding about the function of TAP in altering the electronic performances of FETs. It is found that there are variations in the curves of I_GS_ verse V_GS_ of the as-prepared samples, although they are all in negative values, and the minimum absolute values are located at CNPs. That means that the dominated carriers in forming I_GS_ are electrons, and their quantities are at their minimum at the CNPs, which is in coincident with the basic principles of graphene FETs. As to the shifted curves by adjusting the contents of TAP (i.e., the ratios of *n*), bigger leaking currents (I_GS_) were measured in the samples being pretreated by ultrasonication.

Furthermore, the carriers’ mobilities are evaluated according to the method in the literature [17]; the calculation details are provided in Figure S4. In Figure 2C, it is found that the highest mobilities of holes and electrons are possessed by the reduced GO&TAP (*n* = 4). It is in agreement with TAP-enhanced rGO conductance [11] and porphyrin’s catalysis in HER, which is helpful to accelerate the charge transfer in the channel [13]. However, deviations are still observed here, which include: no increasements could be found when the TAP contents are increased from 1/4 (*n* = 4) to 1/2 (*n* = 2) and from zero (rGO) to 1/8 of GO (*n* = 8).

Generally, the measured electronic features partly agree with the studies in which porphyrin improved highly conductive graphene films [11], but there are still two questions: (1) Provided that porphyrin can speed up the charge transfer between porphyrin and an rGO sheet [13], enhance rGO’s conductivity, and have the potential to catalyze ORR [9], when TAP is added, there should be increased currents (I_DS_) and mobilities from rGO to *n* = 8, and the highest ones should be measured by *n* = 2; but the measured results are not, why? (2) The ultrasonication may generate a cavitation effect and provide a huge impact energy to loosen stacked GO layers and increase the mixing degree between TAP and GO fragments. Then, by the same HHAR, the reduction degree of “ultra_GO&TAP (*n* = 4)” should be higher than “GO&TAP (*n* = 4)”; accordingly, the bigger I_DS_, the higher the carriers’ mobility, and the smaller I_GS_ should be measured by the former one. However, the results are contrary; what is the reason for this?

The answers may come from studying the influences of the TAP mixing degree on rGO’s deoxygenation degree and its lattice recovery. The residue oxygen-containing groups and the distorted positions in the hexagonal carbon planes may serve as the recombination and scattering centers for carriers, as well as lower their mobilities and the output currents. Testification for this deduction is proposed based on the characterization results of Raman, XPS, TEM, and atomic force microscope (AFM) in the following sections.

### 2.2. Characteristic of Raman and XPS

**Figure 3:**
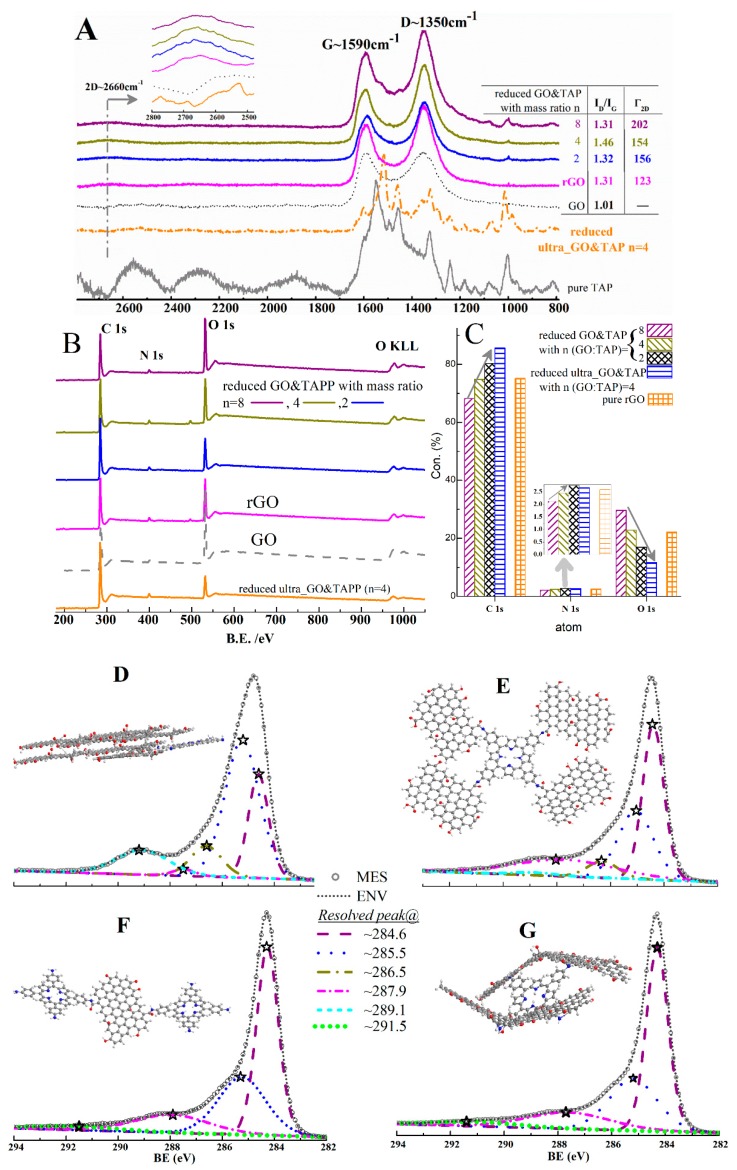
Characterizations of TAP-rGO samples that are the reduced products of GO&TAP (with mass ratios (*n* = GO:TAP) of 8, 4 and 2) and ultrasonically pretreated GO&TAP (ultra_GO&TAP, *n* = 4). (**A**) Raman spectra. (**B**) XPS wide spectra. (**C**) Main elements’ contents. (**D**) to (**G**) are the C 1s core spectra and the X-ray photoelectron spectroscopy (XPS) peak-differentiating analyses for the reduced products of GO&TAP *n* = 8 (**D**), 4 (**E**), and 2 (**F**) and the reduced ultra_GO&TAP (**G**). Data analyses for B to G are executed by CasaXPS Version 2.3.13.

Firstly, it is found in Figure 3A that “reduced GO&TAP” and “rGO” have similar Raman features, including the typical D band, G band, and 2D band which are located at 1350, 1590, and 2660 cm^−1^, respectively. The appearance of 2D peaks in contrast to pure GO and “reduced ultra_GO&TAP” indicates that the planar lattice of rGO nanoflakes are restored in “reduced GO&TAP” and “rGO”, but only to some extent. In other words, the absence of a 2D band in “reduced ultra_GO&TAP” suggests that the sp^2^ two-dimensional carbon network is destroyed, although it has been reduced by the same HHAR. For the reason of this phenomenon, we deduce that since there are no other operations in their preparation procedure, TAP molecules may pierce into GO’s carbon network, being assisted by ultrasonication, via the edge-to-face type π–π* conjugation, which is illustrated by the π–π* satellite peak in Figure 3G.

Moreover, in curves of “rGO” and “reduced GO&TAP”, there are relatively high-intensity ratios of the D peak and the G peak (I_D_/I_G_) and a wide full-width-at-half-maximum (FWHM) of the 2D band (Γ_2D_), as shown by the inserted table in the right side of Figure 2A. Both of them suggest that ruined planar honeycombs exist, and the participation of TAP in HHAR cannot dramatically improve the renovation of GO nanosheets.

Secondly, according to the XPS wide spectra (Figure 3B), the main elements in these samples are C, N, and O; their contents (in Figure 3C) are varied by adjusting components or applying ultrasonic pretreatment to their precursors. Since TAP is the only source that can make the changing of N 1s content, we would like to use it to track the divergence of the TAP mixing degree in these samples. This divergence is found to be in coincidence with the TCCs’ movements in Figure 2B, that is to say: for “reduced GO&TAP”, the growing columns from *n* = 8 to 2 indicate that more N elements were doped, which can induce the right-shifting of their TCCs (dark yellow, blue, and magenta curves in Figure 2B); for “reduced ultra_GO&TAP (*n* = 4)”, its N 1s column’s height is between “reduced GO&TAP *n* = 4” and “*n* = 2”, which verifies our initial deduction that ultrasonication can enhance the TAP mixing degree. Accordingly, there are more N elements in “reduced ultra_GO&TAP” than “reduced GO&TAP”, although they are prepared by same mass ratio (*n* = 4). So far, a mutual corroboration between TCCs (Figure 2B) and XPS data is formed, which demonstrated: there are different TAP mixing degrees in the as-prepared TAP-rGO products, which induced the right-shifted TCCs due to the p-type doping effect [1].

In addition, the C/O ratios are calculated and presented in Figure S5A; it can reflect the different reduction degrees of these samples under the similar HHAR process, while more discussions for them will be presented in the next section, in the light of the above-mentioned questions.

Subsequently, C 1s core spectra analyses are conducted and presented in Figure 3D–G for the samples of “reduced GO&TAP” (*n* = 8, 4, 2) and “reduced ultra_GO&TAP”, respectively. Six peaks can be found in the resolved C 1s spectra, which are listed below [10]: (1) the peaks at about 284.6 eV belong to the carbon atoms in sp2 hybridization, such as C–C, C=C, and C–H; (2) the peaks at about 285.5 eV belong to the sp3 hybridization of lattice carbons, such as C–OH; (3) the peaks at about 286.5 eV are contributed by C=O groups; (4) the peaks at about 289.1 are ascribed to carboxyl (–COOH) groups; and (5) the peaks at about 291.5 eV belong to the π–π* shake-up satellite peak, which is a characteristic of aromatic or conjugated systems [18].

The intensities of these peaks are varied from Figure 3D–G; that is, the intensity of the –N–C(O)– groups in “reduced GO&TAP” are increased from Figure 3D–F; meanwhile, the intensity of the –COOH groups are weakened, even vanished. By comparing with rGO’s C 1s core spectra (Figure S5B), the emerging of amide groups and the fading away of carboxyl groups let us make an imagination about TAP-linked rGO hybrids (as shown by Figure S6 and the insets in Figure 3D–G), according to the literature [12]; that is: under the alkalescent environment of HHAR, a TAP-rGO hybrid can be synthesized based on the condensation of the COOH groups on the edge of GO and –NH_2_ on the substituent groups of TAP. More illustrations for their respective impacts on FET’s electronic features will be presented in Section 2.4.

The appearances of π–π* shake-up satellite peaks in Figure 3F,G indicate that there are increased reduction degrees in the samples of “reduced GO&TAP *n* = 2” and “reduced ultra_GO&TAP *n* = 4”, which are in agreement with the increased C/O ratios given in Figure S5A. This phenomenon is also in accordance with a porphyrin-stabilized GO reduction [11].

### 2.3. Morphology of TEM and AFM

**Figure 4:**
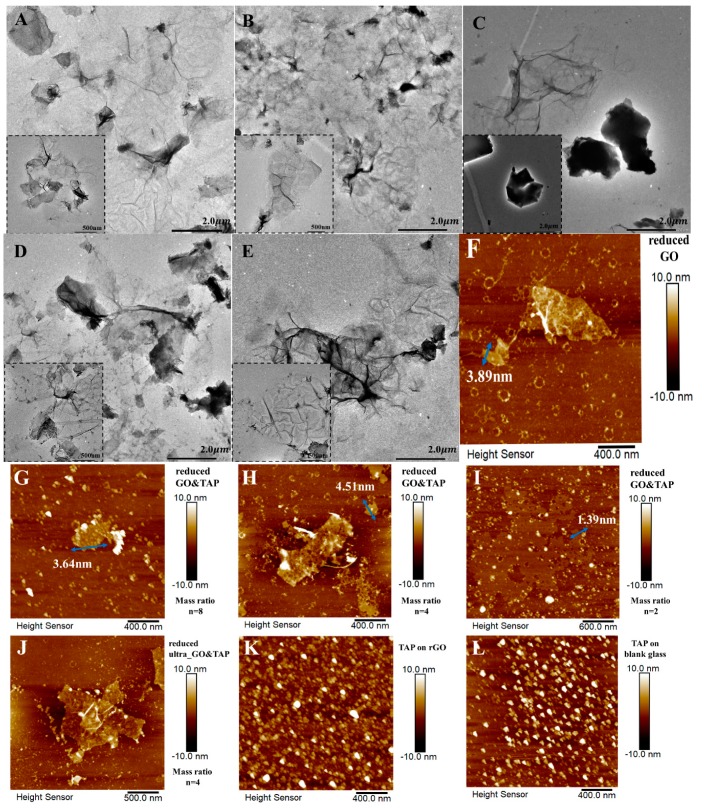
TEM photos of reduced GO&TAP (**A**) and reduced ultra_GO&TAP (**B**), with the same mass ratio of GO:TAP = 4, in comparison with rGO with drop-coated TAP (**C**), together with the photos of reduced GO&TAP with a mass ratio of GO:TAP = 8 (**D**), and 2 (**E**). The thickness of the rGO nanolayers in rGO (**F**) and reduced GO&TAP with different mass ratio of GO:TAP, *n* = 8 (**G**), 4 (**H**), and 2 (**I**) are estimated by using their atomic force microscope (AFM) and the software of NanoScope Analysis 1.5. For comparison, AFM images of reduced ultra_GO&TAP with the mass ratio of GO:TAP = 4 (**J**), drop-coated TAP layer on the rGO-coated (**K**) and the blank glass slides (**L**) are given.

The morphological information is provided by TEM and AFM. Although gossamer-thin layers are observed in all of them (Figure 4A–E), the enlarged scales are found in the images of reduced GO&TAP (*n* = 4, Figure 4A; *n* = 8, 2, Figure 4D,E) and ultra_GO&TAP (*n* = 4, Figure 4B), comparing with pure rGO (Figure 4C). This phenomenon agrees with the amide group-based conjecture, which is proposed in the above section and depicted in Figure 4D,E; that is: the GO platelets are linked by TAP, and after being reduced by HHAR, the larger rGO layers could be observed.

The thickness of these nanolayers is roughly estimated by AFM technology (Figure 4F–I), which are about 3.89, 3.64, 4.51, and 1.39 nm for the rGO and reduced GO&TAP (*n* = 8, 4, 2), respectively. Their layer numbers can be estimated to be 4, 4.7, and 1.5, according to working mode of AFM examination, which is in tapping mode, under which one single graphene layer is about 0.95 nm [19].

In addition, the existence of TAP in reduced GO&TAP can be discriminated by contrasting their microscope with that of pure rGO and TAP. The black dots in Figure 4C (TAP-coated rGO film) indicate that pure TAP molecules tend to accumulate on rGO film; however, a few of the TAP accumulations can be found in the reduced GO and TAP mixtures (Figure 4A,B and Figure 4D,E). Similar phenomena are observed by AFM examinations; that is, the existence of TAP can be discerned by the small bright spots in pure TAP’s AFM (Figure 4K,L), but they are absent from pure rGO (Figure 4F) and sporadically scattered in reduced GO&TAP (Figure 4G–I) and reduced ultra_GO&TAP (Figure 4J).

### 2.4. Approaches of TAP Impacting rGO-FET

**Figure 5:**
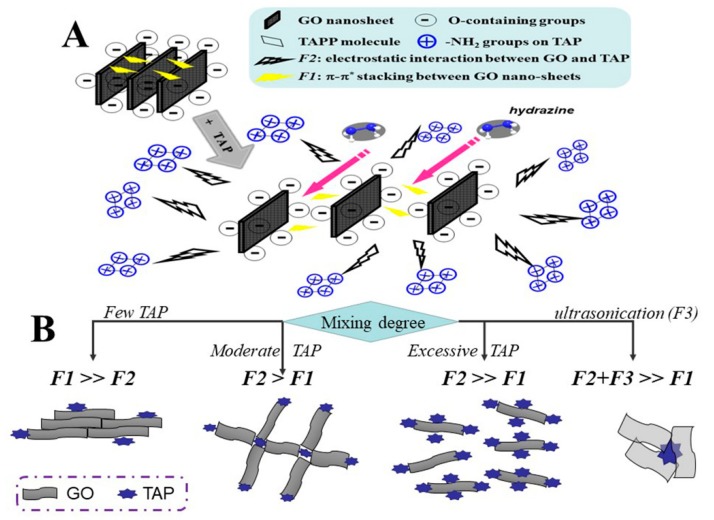
Schematic illustrations for approaches of TAP impacting rGO. (**A**) The interaction between GO and TAP. (**B**) The competition between the GO layers’ π–π* stacking force (*F1*), TAP’s electrostatic attraction to GO (*F2*), and ultrasonication-induced external impact force (*F3*).

In response to the questions presented by the electronic measurements (Figure 2), we deduce that there may be a game relationship amidst GO and TAP mixtures (as diagrammed in Figure 5), that is: depending on the mixing degree which is evidenced by XPS examinations, there will be four competing results of the π–π* stacking force among GO nanosheets (*F1*) against the externally imposed forces, such as the electrostatic attraction between TAP and GO nanosheets (*F2*) and ultrasonication-induced impact force (*F3*). This competing result will decide the deoxygenation degree and the lattice restoring effect, which are ascertained by Figure 3; then, the confusion for the irregular variations in rGO-FETs’ electronic features (Figure 2) could be clarified. More illustrations will be given as follows.

(1) If few TAPs are added (such as GO:TAP = 8), they cannot generate enough force to separate the stacked GO layers (i.e., *F1*>>*F2*), and they tend to attach on the stacked GO layers during HHAR. Though porphyrin has the function to facilitate ORR [9], the amount of TAP is not enough to make an obvious deoxidation effect; on the contrary, the coverage of TAP around the stacked GO layers likely hinders the reducing agent (hydrazine hydrate molecules) arriving at the interlayer of GO and playing its deoxygenation role. This is because the C/O ratio of reduced GO&TAP *n* = 8 is lower than rGO (Figure S5A), which means that the insufficient TAP doping will decrease the reduction degree, and then induce the deteriorated FET features, which are the lowered I_DS_ (marked by the purple arrow in Figure 2A) and the reduced carrier mobilities in comparison with pure rGO-based FET (Figure 2C). So far, the problem (1) could be answered partly.

(2) In the case of moderately added TAP (such as GO:TAP = 4), the stacked GO nanosheets could be detached by the stronger F2 (i.e., *F2*>*F1*), and integrated by the amide linkage (Figure 3E); after being reduced by HHAR, an enlarged rGO-TAP hybrid in a hand-in-hand manner (Figure S6) could be observed by TEM (Figure 4A). Furthermore, the emerging of a 2D band in Raman spectra (Figure 3A) indicates that a more two-dimensional honeycomb lattice (the lamellar structure, as depicted in Figure 1) could be restored in this case than in case (1) and pure rGO. The greater C and less O contents that are found in reduced GO&TAP (*n* = 4) compared with pure rGO (Figure 3C) and the increased C/O ratio (Figure S5A) can also manifest the reduction degree, which in this case is enhanced. All these characterized results point out the graphene-like lattice and increased reduction degree, which can generate the improved FET’s electronic features, such as the enhanced I_DS_, the lowered I_GS_ (the blue solid and dash curves in Figure 2A), and the highest carrier mobilities in Figure 2C.

Furthermore, if TAP is overmixed with GO by excessive TAP molecules (for instance, GO:TAP = 2) or ultrasonication, the stacked GO layers can be more deeply separated via two pathways, as depicted in Figure 5 and illustrated below.

(3) If the amount of TAP is sufficient enough to not only generate stronger *F2* (*F2* >> *F1*), which can rip off the stacked GO layers, but also make each of the dispersed GO fragments enclosed by TAP molecules by amide linkages or an aromatic π–π* interaction (evidenced by XPS analysis in Figure 3F). In this state, the surrounded TAP molecules on GO fragments will not hamper HHAR such as in case (1), because the GO lamellae have been detached, rather than the stacked one in case (1). That may be the reason for the continuously increased C/O ratio (Figure S5A) and the typical rGO’s Raman spectra (Figure 3A). Its TEM (Figure 4E) and AFM (Figure 4I) images also indicate they have acceptable morphological features. However, the fragmental rGO sheets in this case cannot provide the same smooth channel as case (2), which may induce the lowered I_DS_ values in Figure 2A, as well as the decreased carrier mobilities in Figure 2C.

(4) For the same mass ratio of GO:TAP (*n* = 4), if ultrasonication is applied, it may generate a cavitation effect and provide an extra force (*F3*) with huge impact energy to loosen stacked GO layers and make an overmixing effect, and after suffering HHAR, TAP and rGO nanosheets will be in tangles (inset of Figure 3G). This tangled hybrid (the Tangled structure, as depicted in Figure 1) can be identified by its Raman spectrum (the orange curve in Figure 3A) and the abnormal contents of C and N in Figure 3C (the highest C column corresponding to the relative lower N column in contrast to reduced GO&TAP (*n* = 2)). Although the deoxygenation degree can be augmented in this tangled TAP-rGO hybrid (confirmed by its C/O ratio in Figure S5A), the desired lamellar graphene-like lattice cannot be obtained, as evidenced by the absence of a 2D band in its Raman spectrum.

Therefore, the answer for the second problem, which is put forward for the lowered I_DS_ and increased I_GS_ (magenta curves in Figure 2) might be: in the tangled TAP-rGO, carriers cannot be confined in the horizontal direction (Figure S1) as they are in lamellar TAP-rGO and rGO. Moreover, when fostered by the HER effect [13] and driven by the vertical voltage (V_GS_), many of them will escape from the channel region and form the leaking current I_GS_. Correspondingly, less carriers can be left in the channel region to form I_DS_. As a result, the increased I_GS_ and decreased I_DS_ are measured.

## 3. Discussion

In conclusion, a study about TAP’s roles in altering rGO-FET electronic features (including the output currents I_DS_, the leaking currents I_GS_, and the carriers’ mobilities) is conducted from different points of view. The problems in the influences of TAP on rGO-FET are posed based on the electronic examinations, at first; then, the surmised answer is proposed and tested by the comprehensive analysis of characterization results from Raman, XPS, TEM, and AFM. It is found that TAP can interfere with HHAR’s reduction degree, depending on its contents and the overmixing pretreatment. That is, TAP can improve or impair the reduction degree and rGO’s morphology, and ultimately determine rGO-FET’s electronic features, depending on the competition between the GO nanosheets’ natural stacking force (*F1*) and external detaching forces (*F2* or *F3*). For rGO-FETs’ demand of higher mobility and the preservation of oxygen-groups in rGO, the moderately doped and without extra ultrasonic pretreated TAP-rGO hybrid could provide a promising solution. We expect that the observations and deliberations in this work can provide some helpful and constructive recommendation for producing low-cost chemically converted graphene and promoting its applications in bioFETs or other kinds of components.

## 4. Materials and Methods

### 4.1. Materials

The main chemicals in the preparation of GO are: graphite powder (Beijing HWRK Chem. Co. Ltd., Beijing, China), H_2_SO_4_ (98.0%), hydrogen peroxide (H_2_O_2_, 30.0%), and permanganate (KMnO_4_, 99.5%) (Tianjin Chemical Reagent wholesale company, Tianjin, China). The chemical reagents used in the reduction process of GO are ammonia solution (25%, Tianjin wind boat chemical reagent Technology Co. Ltd., Tianjin, China), hydrazine hydrate (80%, Tianjin Fuyu Fine Chemical Co. Ltd., Tianjin, China) and TAP (5, 10, 15, 20-(tetra-4-aminophenyl) porphyrin, 95%, Hangzhou Expo biotech Co. Ltd., Hangzhou, China). Conductive silver paste (Shenzhen SYREO electronic paste Ltd., Shenzhen, China), glutaraldehyde (GA, Chengdu Huaxia Chemical Reagent Co. Ltd., Chengdu, China), (3-Aminopropyl) triethoxysilane (APTES, Sigma-Aldrich, Saint Louis, MO, USA), and *N*,*N*-dimethyl formamide (DMF, J&K Scientific, Beijing, China)are used for device fabrication and sample preparation for characterization.

The main reagents used in the experiments are: (1) Piranha solution is prepared by 30 mL of H_2_SO_4_ (98.0%) and 10 mL of H_2_O_2_ (30.0%). (2) APTES is diluted in deionized water (DIW) with the volume ratio (*v*/*v*) of 1:10, pH = 7.4. (3) GA is dissolved in DIW (*v*/*v* = 1:20), pH = 7.4. (4) The phosphate-buffered saline (PBS) is Na_2_HPO_4_ and NaH_2_PO_4_ in DIW with the concentration of 100 mM, and pH is adjusted by HCl at about 7.4.

### 4.2. Syntheses of GO

GO is prepared according to the modified Hummers method, which was used in our previous work [20]; the operations are outlined as follows. (1) The ground graphite powder is treated by H_2_SO_4_ (98%) and stirred for 1 h. (2) KMnO_4_ is gradually added into the mixture under the ice bath condition and stirred for 1 h. (3) H_2_O_2_ (30%) is added in the mixture after the above two steps to remove the potential impurities. (4) The solid substance in suspension is precipitated and dried to obtain the powder of graphite oxide. (5) This powder is suspended in DIW with the concentration of 0.25 mg/mL. Then, after suffering centrifugation, its supernatant is extracted, which is the GO dispersion waiting to be reduced by the following HHAR, as illustrated in Figure 1.

### 4.3. Reduction Precursor and Reduction Process

There are three kinds of reduction precursors: pure GO, its mixture with TAP (GO&TAP), and the ultrasonically treated GO&TAP (ultra_GO&TAP). GO&TAP is prepared by adding TAP into GO dispersions with the mass ratio of GO:TAP (*n*) = 8, 4, 2. Ultra_GO&TAP is obtained by treating the mixture of GO&TAP (*n* = 4) with ultrasonication in a water bath for 2 h.

The reductions for GO and its different mixtures with TAP are conducted by the similar operations, which are: (1) adding ammonia water to 30 mL of the reduction precursor to adjust the pH to approximately 10–11; (2) adding about 21 μL of hydrazine hydrate as a reducing agent and shaking; (3) shaking the mixture for 1 h under the water-bath condition of 98 °C; then finally, (4) after being treated by low-speed centrifugation, the reduced product is obtained from the supernatant of the mixture.

### 4.4. Device Fabrication

The rGO-FETs are fabricated on the thoroughly cleaned glass substrates following the similar procedure in the previous work [2]. The main operations are outlined here. (1) The substrates are immersed in the APTES solution for 2 h at 50 °C and immersed in the GA solution for 1 h at room temperature. (2) After being rinsed with DIW, they are drop-coated by rGO, reduced GO&TAP, or reduced ultra_GO&TAP. (3) After being completely rinsed and dried, conductive silver paste is drawn as the patterns in the inset of Figure 2A and baked in an oven (60 °C, 5 h) to form source (S) and drain (D) contacts. With the formation of S and D, the channel geometry can be defined, which is 3 mm in length and 18 mm in width. (4) The insulating ink is spread on the electrodes of S and D after bonding the conducting wires, only the channel region is left being uncovered and directly exposed to the electrolyte solution (PBS in this work). (5) A cover plate is fixed on the top of the prepared devices to make the electrode gate (G) suspended on the channel film, as depicted in the inset of Figure 2A. The cross-section of the cover plate is depicted in Figure S7, in which the platinum plate electrode (used as G) is adhered to the substrate of a polyethylene terephthalate (PET) thick plate (5 mm).

### 4.5. Apparatus

Dimension Icon (Bruck, Billerica, MA, USA) and JEM-2800 (JEOL Co. Ltd., city, province, Japan) are used for taking the images of atomic force microscope (AFM) and transmission electron microscope (TEM). Raman spectra are examined by RTS-HiR-AM (Titian Electro-Optics Co. LTD, Hong Kong, China). XPS examinations are conducted by Axis Ultra DLD (Kratos Analytical Ltd., Manchester,UK). A digital source meter (DSM) B2900A (Agilent Technology Co. Ltd., Palo Alto, CA, USA) was used for measuring the electronic features of FETs and FETs’ electrodes of D, S, and G to DSM, as depicted in the inset of Figure 2A.

## Supplementary Materials

The following are available online. Figure S1: The definition of voltage directions for rGO-FETs, Figure S2: The unnormalized transfer curves of the prepared FETs, Figure S3: The gate leakage of the FETs with different reduced product, Figure S4: The calculated conductance verse carriers’ concentration, Figure S5: XPS analyses, Figure S6: The schematic structure of TAP and rGO integration, Figure S7: The schematic depiction of the cover plate for the gate electrode.
