# Supplementary material for: Function of Tetra (4-Aminophenyl) Porphyrin in Altering the Electronic Performances of Reduced Graphene Oxide-Based Field Effect Transistor
Source: Molecules. 2019 Oct 31;24(21):3960. doi: 10.3390/molecules24213960 (PMC6865170; doi:10.3390/molecules24213960)
Supplement: Supplementary file 1 [file molecules-24-03960-s001.pdf]

Supplementary Materials

Function of Tetra (4-Aminophenyl) Porphyrin in Altering the Electronic Performances of Reduced Graphene Oxide-Based Field Effect Transistor

Shihui Hu and Yunfang Jia *

College of Electronic Information and Optical Engineering, Nankai University, Tianjin,300350 , China; 1120170101@mail.nankai.edu.cn

* Correspondence: jiayf@nankai.edu.cn

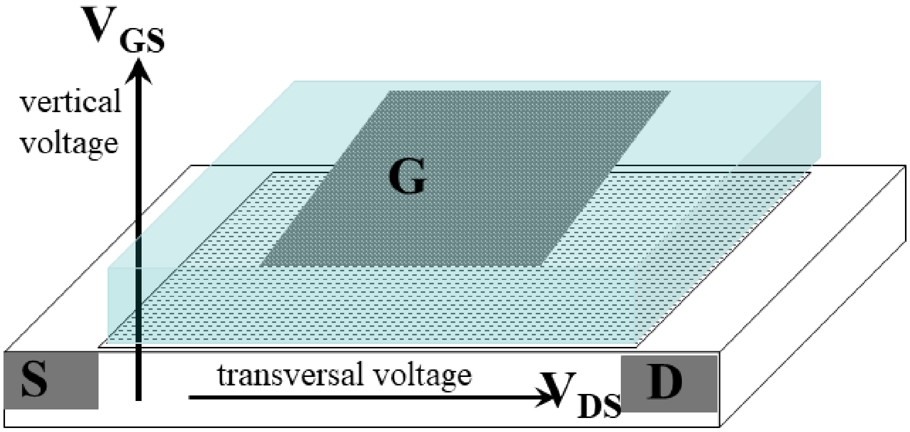

Figure S1. The definition of voltage directions for rGO-FETs.

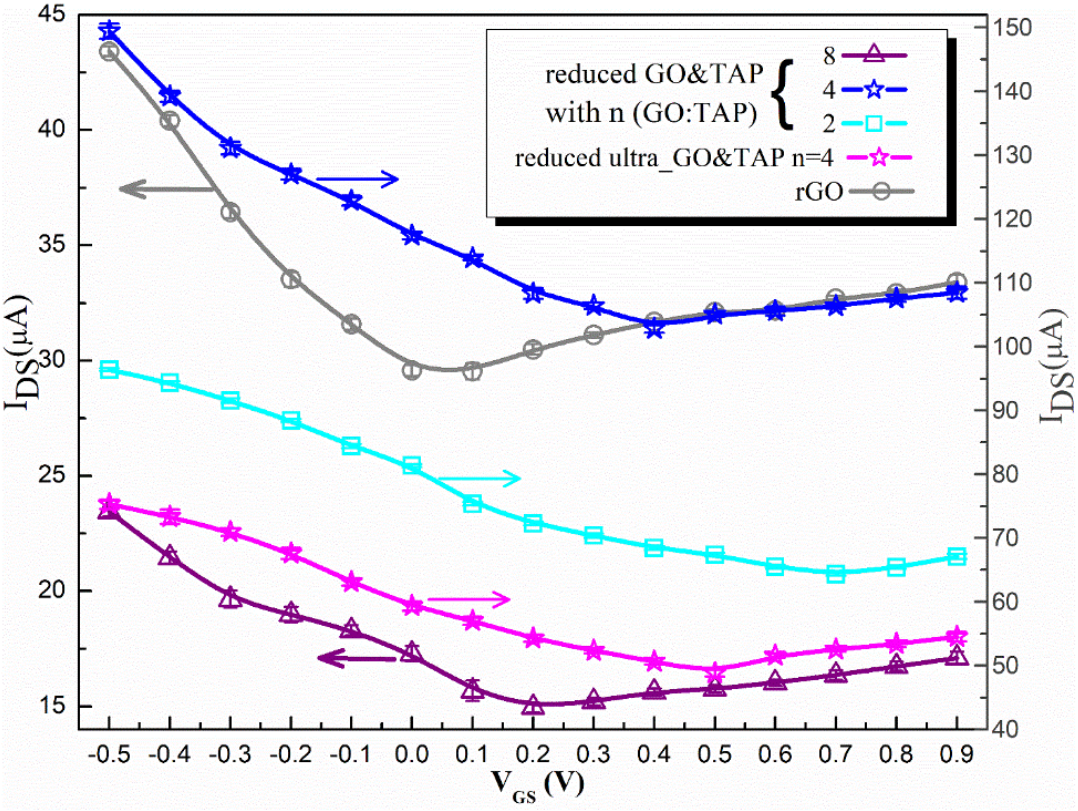

Figure S2. The unnormalized transfer curves for the preapred FETs based on different reduced products which are the reduced GO and TAP mixture (reduce GO&TAP with mass ratio of n (GO: TAP) = 8, 4, 2), the reduced ultrasonically treated GO&TAP (reduced ultra_GO&TAP, n = 4), as well as pure rGO. In which, the voltages of V_{GS} are changed from -0.5 to 0.9, and V_{DS} is controlled at 0.5V.

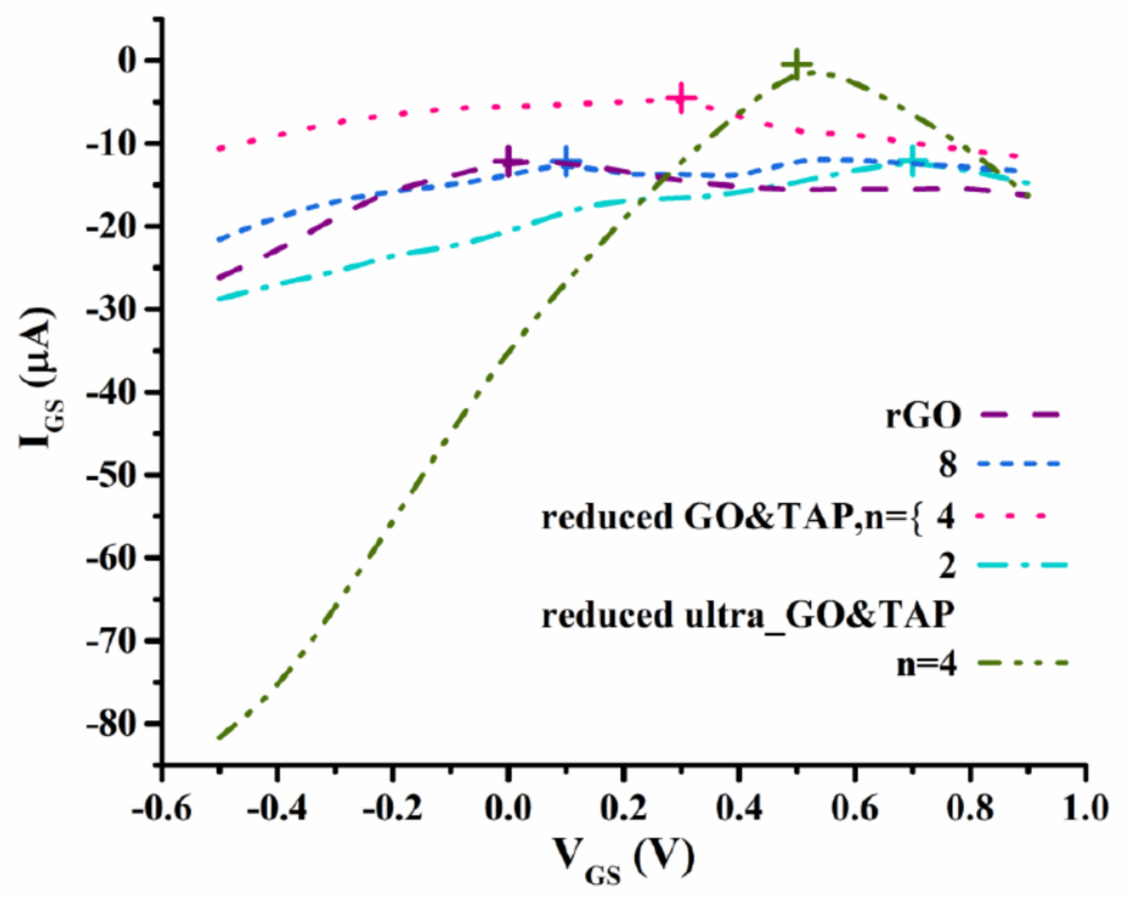

Figure S3. The relations of leakage currents (I_{GS}) with the voltages of V_{GS}, when V_{DS} is controlled at 0.5 V.

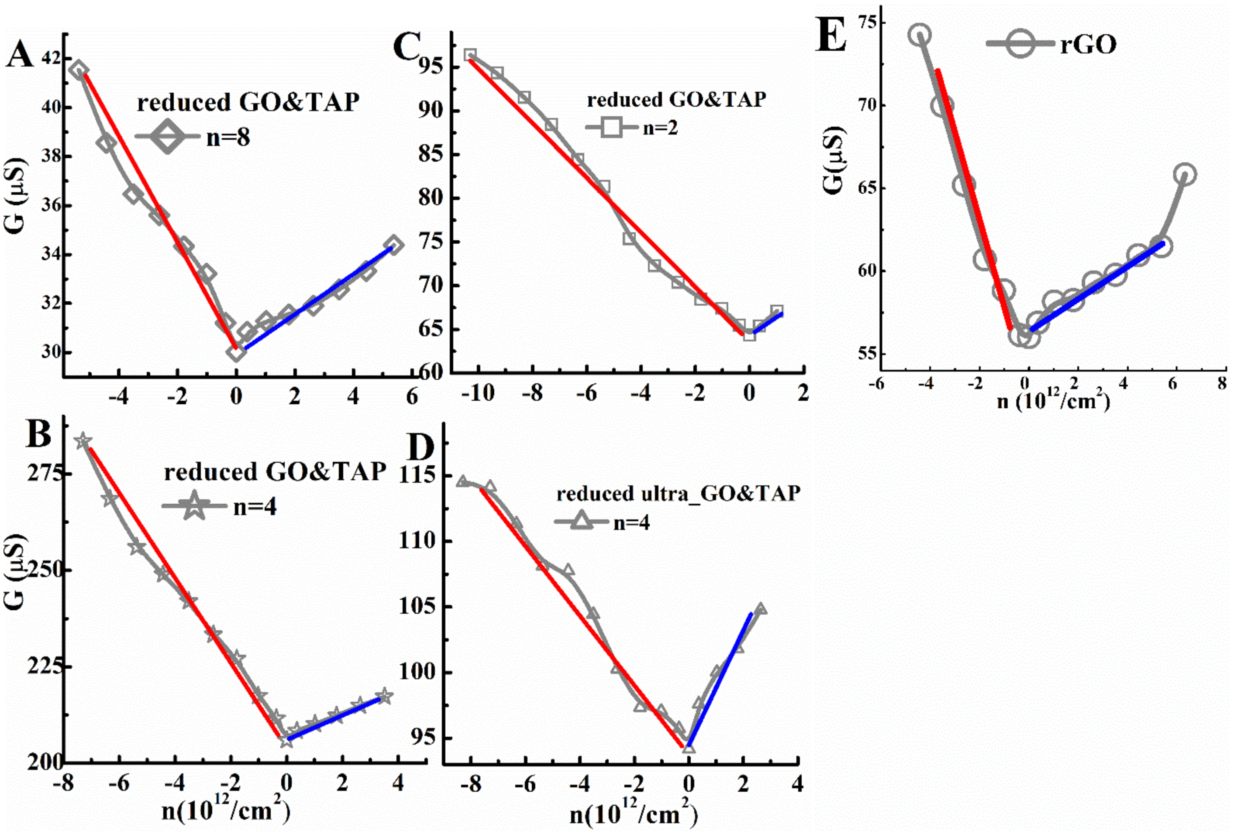

Figure S4. The calculated conductance verse carriers' concerntration curves for the reduced GO&TAP n=8 (A), 4 (B), 2(C) . Based on these data, the carrier mobility can be calculated, the results are presented in **Figure 1**C. The calculation for them is outlined in here [18]:

Firstly, n is calculated by the following formula:

$$|V_{ref}\text{-}V_{CNP}|=\frac{hV_F\sqrt{\pi n}}{e}+\frac{e\,n}{C_{DL}} \quad (1)$$

in which h is Planck constant, V_F is Fermi velocity (10^6 m/s), C_{DL} is the capacitance of EDL (2 μF/cm^2).

Second, the conductivity is derived from voltage and current, according to equation (2)

$$G=\frac{\delta I_{ds}}{\delta V_{ds}}\,(S) \quad (2)$$

The calculated G and n are presented in **Figure S2.**

Thirdly, the carrier mobility is calculated based on the equation (3),

$$\mu=\frac{\delta G}{\delta n}\,(cm^2/V\cdot s) \quad (3)$$

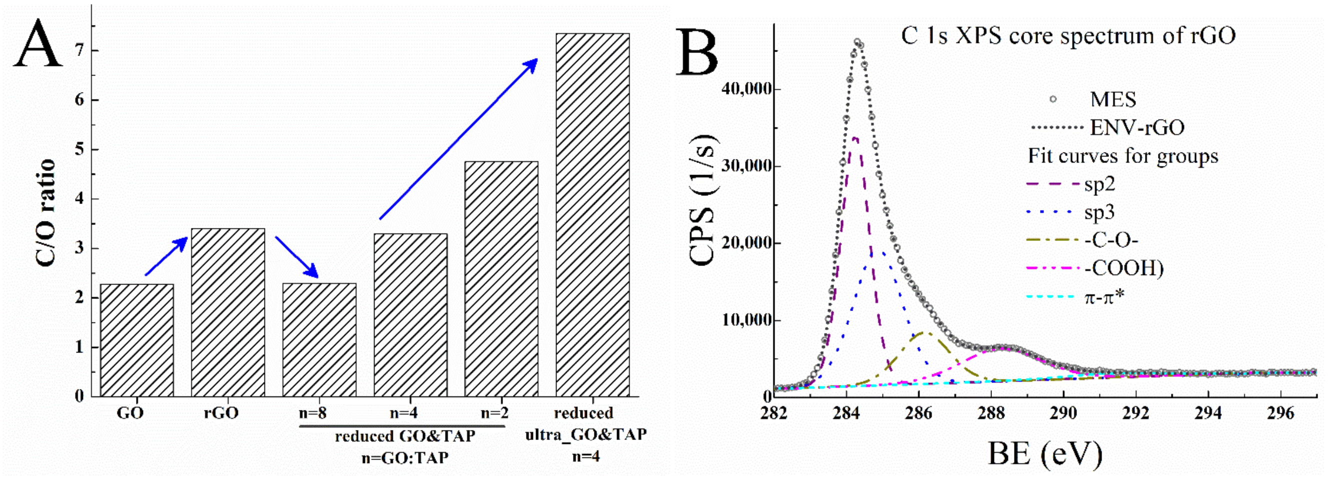

Figure S5. **A**, the quantifications of C/O ratios for the samples of GO, rGO, reduced GO&TAP (n = 8, 4, 2) and the reduced ultra_GO&TAP (n = 4). **B**, XPS C 1s core spectrum of rGO and the fit curves.

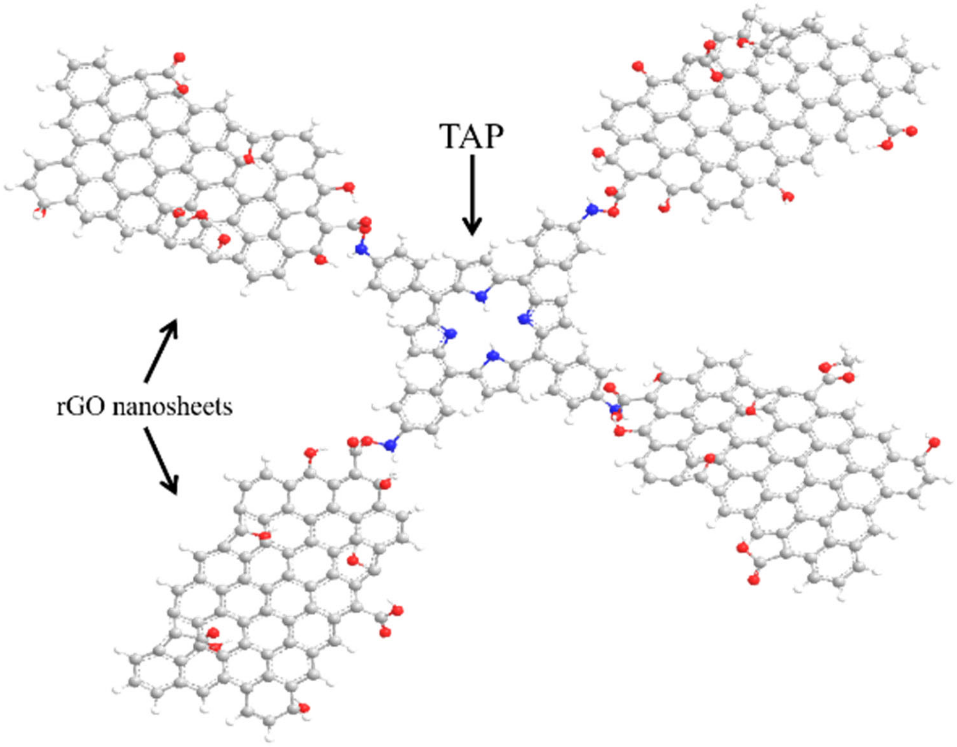

Figure S6. The schematic structure of TAP and rGO integration in a hand-in-hand manner, that is: the amide linkage is formed between -NH_3 groups around tetrapyrrole and COOH groups at the edge of GO, after being reduced, it is turned to rGO-TAP hybrid.

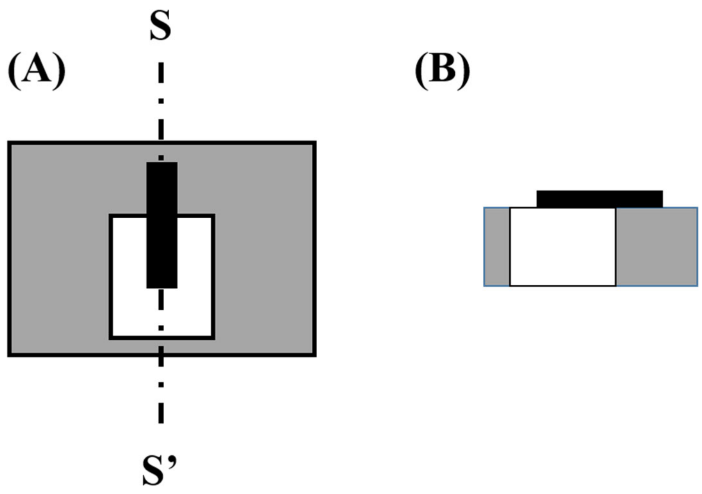

Figure S7. The schematic depiction of the cover plate for the gate electrode. In which, (A) is its top-view, (B) is the cross-section along SS'.
